# Immune dysregulation in endometrial tuberculosis: elevated HLA-G and IL-1Ra as key modulators

**DOI:** 10.3389/fcimb.2025.1548238

**Published:** 2025-05-01

**Authors:** Dan Huang, Jinlong Dai, Haotian Yu, Wen Chen

**Affiliations:** ^1^ Graduate School of Hebei North University, Zhangjiakou, China; ^2^ Department of Reproductive Medicine Clinic, The Eighth Medical Center, Chinese PLA General Hospital, Beijing, China; ^3^ Department of Pathology, The Eighth Medical Center, Chinese PLA General Hospital, Beijing, China

**Keywords:** endometrial tuberculosis, pulmonary tuberculosis, pathological characteristics, HLA-G, tuberculous granuloma, IL-1ra

## Abstract

Endometrial tuberculosis (ETB) is a reproductive system infection caused by Mycobacterium tuberculosis, primarily invading the endometrium through hematogenous dissemination. This study included 10 patients diagnosed with ETB and 10 patients with pulmonary tuberculosis (PTB) to analyze their clinical, pathological, and immunological characteristics. Anatomically, PTB presented the highest prevalence among tuberculosis cases. Compared to PTB imaging, CT scans of ETB showed less distinctive diagnostic features. Pathologically, abscess formation was more frequently observed in ETB patients than in PTB patients, suggesting a more intense local inflammatory response in ETB. However, there were no statistically significant differences in granulomatous lesions, caseous necrosis, coagulative necrosis, inflammatory necrosis, exudation, acute inflammation, or fibrous tissue hyperplasia between the two groups. Immunohistochemical analysis revealed higher infiltration of macrophages (CD68) in ETB lesions compared to PTB, whereas the counts of T cells (CD3+, CD4+, CD8+) and B cells (CD20) showed no significant differences. Notably, the expression levels of HLA-G and IP-10 were significantly elevated in the lesion areas of ETB compared to PTB. Similarly, the expression of HLA-G, IP-10, IL-1Ra, and IL-10 was significantly higher in the ETB group than in the PTB group. Furthermore, HLA-G and IL-1Ra expression levels were markedly elevated in ETB lesion areas compared to surrounding normal endometrial tissue. HLA-G plays a pivotal role in immune tolerance by modulating local immune responses, while IP-10 is involved in chronic inflammatory signaling. IL-1Ra and IL-10 are key regulators of endometrial immune homeostasis, counterbalancing inflammatory responses that could otherwise disrupt reproductive function. These immunoregulatory factors are crucial in maintaining immune tolerance within the endometrium and may influence immune responses associated with endometrial tuberculosis.

## 1 Introduction

Endometrial Tuberculosis (ETB) is a reproductive system infection caused by Mycobacterium tuberculosis, primarily transmitted hematogenously to the endometrium (Abbara and Davidson, 2011). Globally, the incidence of tuberculosis has been steadily declining (Global, regional, and national age-specific progress towards the 2020 milestones of the WHO End TB Strategy: a systematic analysis for the Global Burden of Disease Study 2021, 2024). Although the prevalence of ETB is relatively low, it significantly impacts women’s reproductive health and is recognized as a leading cause of infertility in affected populations (Sharma et al., 2008). It is estimated that in countries with high tuberculosis prevalence, approximately 5-16% of infertility cases are associated with genital tuberculosis, with ETB being the predominant subtype. ETB directly impairs the function and structure of the endometrium, resulting in menstrual irregularities, infertility, and other reproductive health issues (Bhanu et al., 2005).

Despite its significant impact on female reproductive health, the diagnosis of endometrial tuberculosis (ETB) remains highly challenging. Existing literature indicates that the clinical presentation of ETB often lacks specificity, making it easily confounded with other gynecological conditions (Sharma et al., 2021; Tzelios et al., 2022). Current imaging modalities, including CT and MRI, demonstrate limited sensitivity in identifying ETB, further complicating clinical diagnosis (Xiang et al., 2021). Emerging technologies such as multimodal imaging and ultrasound texture analysis show promise in enhancing diagnostic sensitivity and specificity (Garg et al., 2021). Histopathological examination remains the gold standard for TB diagnosis, with characteristic features including chronic granulomatous inflammation and caseous necrosis (Rammeh and Romdhane, 2022). Unlike other tissues, the endometrium undergoes cyclic changes, including proliferative, secretory, and menstrual phases. Melkamu K et al. reported that ETB commonly presents with early granulomas and a low acid-fast bacilli positivity rate (Melkamu et al., 2024).

Infertility is a reproductive issue affecting millions of couples worldwide, with its occurrence attributed to various factors, including hormonal imbalances, tubal obstruction, and ovarian dysfunction (Carson and Kallen, 2021; Concepción-Zavaleta et al., 2023). Additionally, immune system dysregulation, particularly abnormalities in cellular immunity, is also considered one of the significant causes of infertility (Sen et al., 2014).The immune response triggered by Mycobacterium tuberculosis infection stimulates cellular immunity, leading to the proliferation of T cells and the release of various cytokines to recognize and eliminate the infection (de Martino et al., 2019). The pathogenesis of ETB involves a complex interplay between Mycobacterium tuberculosis and the host immune system, leading to chronic inflammation, fibrosis, and endometrial dysfunction (Sharma et al., 2023). However, the immunological mechanisms underlying ETB pathogenesis remain inadequately understood. ETB is characterized by a dysregulated immune response, involving macrophage activation, altered CD4+/CD8+ T-cell balance, and aberrant cytokine production, which collectively contribute to endometrial fibrosis and dysfunction. Macrophages play a dual role in both host defense and immune regulation in ETB, while the CD4+/CD8+ T-cell balance is crucial in maintaining reproductive immunotolerance and pathogen clearance (Rao et al., 2015; Scriba et al., 2017; Lv et al., 2023).These immune cell subsets are particularly relevant in reproductive health, as disruptions in their function can lead to implantation failure, recurrent pregnancy loss, and infertility (Gupta and Gupta, 2020). A comprehensive understanding of ETB-related infertility requires an integrated approach that considers immunological, histopathological, and clinical perspectives, given the complex interplay between host immune responses and endometrial function. By investigating the immunoregulatory factors associated with ETB, this study aims to elucidate their potential roles in disease pathogenesis and reproductive outcomes, thereby contributing to improved diagnostic and therapeutic strategies.

## 2 Materials and methods

### 2.1 Specimen collection

This study is a retrospective analysis of patients diagnosed with ETB. Tissue samples were obtained through surgical curettage during the secretory phase of the menstrual cycle, prior to tuberculosis treatment. The participants’ ages ranged from 20 to 65 years. The medical records analyzed in this study were collected from inpatients at the Eighth Medical Center of the PLA General Hospital between January 2013 and October 2024. The hospital is a comprehensive, tertiary grade A hospital in Beijing, with particular expertise in the diagnosis and management of tuberculosis. The inclusion criteria for the case group (ETB) were as follows: female patients aged 18 or older, infertility diagnosed clinically, defined by the World Health Organization as the inability to conceive after 12 months of regular unprotected intercourse, menstrual irregularities, or chronic pelvic pain, and pathologically diagnosed with ETB; and no prior history of antituberculous treatment. The exclusion criteria for the case group (ETB) were as follows: the presence of other infectious diseases, malignancies, severe hematological disorders, or autoimmune diseases; a history of other known causes of infertility, such as polycystic ovary syndrome, tubal obstruction, and others. The inclusion criteria for the control group (PTB) were as follows: female patients with PTB without evidence of genital involvement. The exclusion criteria for the control group (PTB) were as follows: the presence of other infectious diseases, malignancies, severe hematological disorders, or autoimmune diseases.

### 2.2 Methods

#### 2.2.1 Imaging examination

In this study, CT scans were employed to evaluate both endometrial tuberculosis (ETB) and pulmonary tuberculosis (PTB). The advantage of CT lies in its ability to provide detailed assessments of endometrial morphology and the extent of involvement, enabling the evaluation of lesion activity. These capabilities are particularly valuable for the early diagnosis, differential diagnosis, and assessment of treatment efficacy in ETB (Shibuki et al., 2024; Yayan et al., 2024).

#### 2.2.2 H&E staining

The endometrial tuberculosis and pulmonary tuberculosis tissue samples were fixed in 4% paraformaldehyde for 24 hours to preserve tissue structure and cellular morphology. After dehydration through a graded series of 80%, 95%, 95%, 100%, 100%, and 100% anhydrous ethanol, the samples were embedded in paraffin to harden the tissue for sectioning. The paraffin-embedded tissues were sliced into 3-μm-thick sections and mounted onto glass slides. The slides were then baked at 72°C for 30 minutes to ensure the tissue sections adhered firmly to the slides. Paraffin was removed using xylene, followed by gradient ethanol washes to eliminate residual paraffin and prepare the slides for staining.For hematoxylin and eosin (H&E) staining, the slides were stained with hematoxylin for 30 seconds to stain the nuclei blue-purple. Subsequent steps included differentiation in hydrochloric acid, bluing with ammonia water, counterstaining with eosin for 5 seconds, dehydration with gradient ethanol, and clearing with xylene. Finally, the slides were mounted with neutral resin. The H&E-stained sections were examined under a microscope to observe basic histological features and cellular structures.

#### 2.2.3 Acid-fast staining

Each samples section(3um) were made and mounted onto glass slides. The slides were baked at 72°C for 30 minutes to ensure firm adherence of the tissue sections. Deparaffinization was performed twice using xylene, with each step lasting 10 minutes, to remove residual paraffin. The sections were sequentially washed with 100%, 90%, and 80% anhydrous ethanol (gradient ethanol), with each wash lasting for 5 minutes, in preparation for staining.Next, 2–3 drops of carbol fuchsin solution were applied to the sections, which were stained for 2 hours to achieve coloration. The sections were then decolorized with 1% acid alcohol until they exhibited a pale pink appearance. Hematoxylin staining was performed for 30 seconds to impart a blue-purple color to the nuclei. Differentiation with 10% hydrochloric acid and bluing with ammonia water were conducted to enhance the contrast and clarity of the nuclei. The sections were dehydrated again using gradient ethanol and cleared with xylene to render them transparent. Finally, the slides were sealed with neutral resin to protect the sections.

#### 2.2.4 Identification of mycobacterium

**Figure 1:**
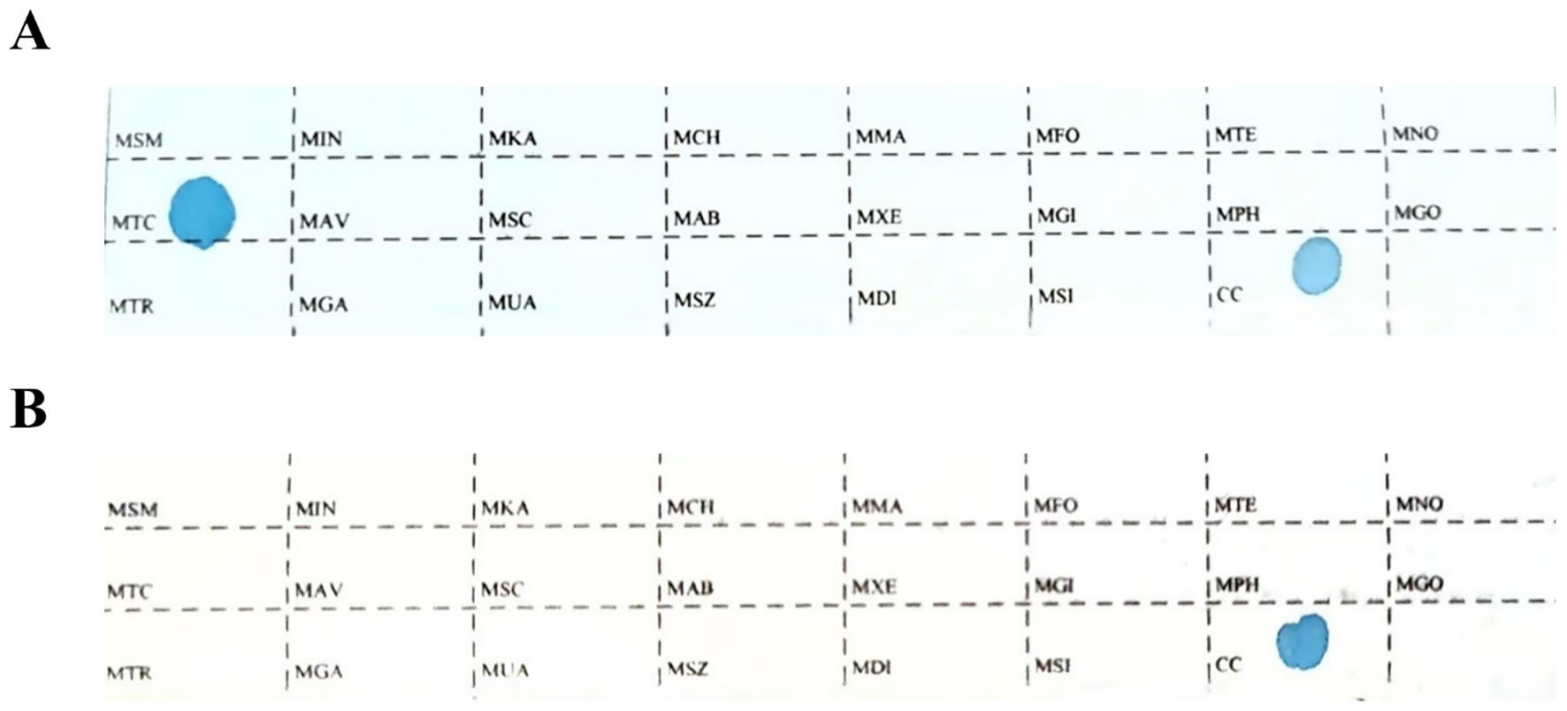
The sequence of detection loci on the membrane strip. MTC: Mycobacterium tuberculosis complex; CC: quality control locus; all other loci represent non-tuberculous mycobacteria detection sites. **(A)** The positive Mycobacterium control. **(B)** The negative Mycobacterium control.

Tissue specimens from each patient were sectioned into 8–10 slices, each with a thickness of 5–10 μm, and placed in 1.5 mL centrifuge tubes for deparaffinization, lysis, digestion, and DNA extraction. DNA was isolated using a gene detection kit (Shenzhen Yaneng Biosciences Co., Ltd., China), and mycobacterial species were identified in PCR tubes. For each reaction, 4 μL of extracted DNA was added to the PCR tube for amplification.After DNA amplification, the membrane strips and amplified products were transferred to a test tube containing 5–6 mL of Solution A and heated in a boiling water bath for 10 minutes to ensure complete reaction. The membrane strips and products were then hybridized at 59°C for 1.5 hours to facilitate the binding of DNA to the probes on the strips. Following hybridization, the strips were washed to remove nonspecific bindings and incubated in Solution A containing peroxidase (POD) enzyme for 30 minutes to promote the color development reaction. Subsequently, the strips were placed in a color development solution for 10 minutes, during which the appearance of blue dots indicated the detection of target loci.The reaction was terminated by rinsing the strips with purified or deionized water. The presence and position of blue dots on the strips corresponded to different detection loci. Positive and negative controls were included in each experiment to ensure the accuracy and reliability of the results (**Figure 1**).

#### 2.2.5 Immunohistochemical staining

Sections of 3 μm thickness were cut from each sample and mounted onto glass slides. The slides were baked at 72°C for 30 minutes to ensure firm adherence of the tissue sections. The slides were then placed in an automated immunohistochemistry (IHC) staining machine (Benchmark XT, Ventana Medical Systems Inc., Tucson, USA), and a pre-programmed staining protocol was initiated. During the staining process, the primary antibody was manually applied to specifically bind to the target antigen. Subsequently, the slides were washed, followed by hematoxylin staining for 10 seconds, differentiation with hydrochloric acid, and bluing with ammonia water to enhance nuclear staining. The sections were then dehydrated using gradient ethanol and cleared with xylene to facilitate microscopic examination. Finally, the slides were sealed with neutral resin.

### 2.3 Materials and antibodies

The antibodies used in this study are as follows: CD3 (RabMAb, ZA-O503), CD4 (mouse monoclonal antibody, ZM-0418), CD8 (RabMAb, ZA-0508), CD20 (mouse monoclonal antibody, ZM-o039RUO), and CD68 (mouse monoclonal antibody, ZM-0o60), all purchased from OriGene Technologies, China. The antibody panel used in this study was employed to assess the key immune cell populations involved in the pathogenesis of ETB. CD3, CD4, and CD8 were used to identify T cell subsets crucial for immune regulation. The macrophage marker CD68 was used to detect macrophage infiltration, a key feature of granulomatous inflammation. CD20, a marker of B cell activity, was used to evaluate the extent of involvement of humoral immune responses. HLA-G (RabMAb, ab283260), IP-10 (RabMAb, ab306587), IL-1Ra (RabMAb, ab303490), and IL-10 (rabbit polyclonal antibody, ab217941) were purchased from Beijing XinSaiMei Biotechnology Co., Ltd. Given their roles in immune tolerance and inflammation, these immunoregulatory markers were selected to explore the pro-inflammatory and anti-inflammatory balance in ETB.

### 2.4 Staining results judgment

The interaction between antibodies and antigens exhibits high specificity, which forms the basis of immunohistochemistry (IHC) for the localization, qualitative analysis, and relative quantification of specific antigens in tissues or cells. Under microscopic observation, the presence of specific yellow or brown granules indicates a positive reaction. This technique evaluates the intensity of immune responses by quantifying positive cells marked by specific antibodies such as CD3, CD4, CD8, CD20, and CD68.For other antibodies in this study, such as HLA-G, IP-10, IL-1Ra, and IL-10, staining intensity was categorized as “0,” “1+,” “2+,” “3+,” or “4+.” A score of “0” indicates a negative result, where no staining is observed, suggesting the absence of the antigen in the sample. A score of “1+” denotes weak positivity, reflecting low protein expression levels, with light yellow staining visible under the microscope. A score of “2+” represents positivity, characterized by moderate staining that appears brown under the microscope. A score of “3+” indicates strong positivity, with more pronounced staining visible as dark brown. Finally, a score of “4+” signifies extremely strong positivity, indicating high protein expression with widespread deep brown or brownish-black staining. In summary, IHC leverages variations in staining intensity to assess protein expression levels in biological samples.

### 2.5 Statistical analysis

Statistical analysis was performed using SPSS software. Fisher’s exact test was applied to compare incidence rates between the two groups, while t-tests were conducted to assess differences in cell counts. A p-value of less than 0.05 was considered statistically significant. Additionally, the Mann-Whitney U test was used to evaluate differences in staining intensity for various markers between the pulmonary tuberculosis group and the endometrial tuberculosis group.

## 3 Results

### 3.1 Clinical data

**Figure 2:**
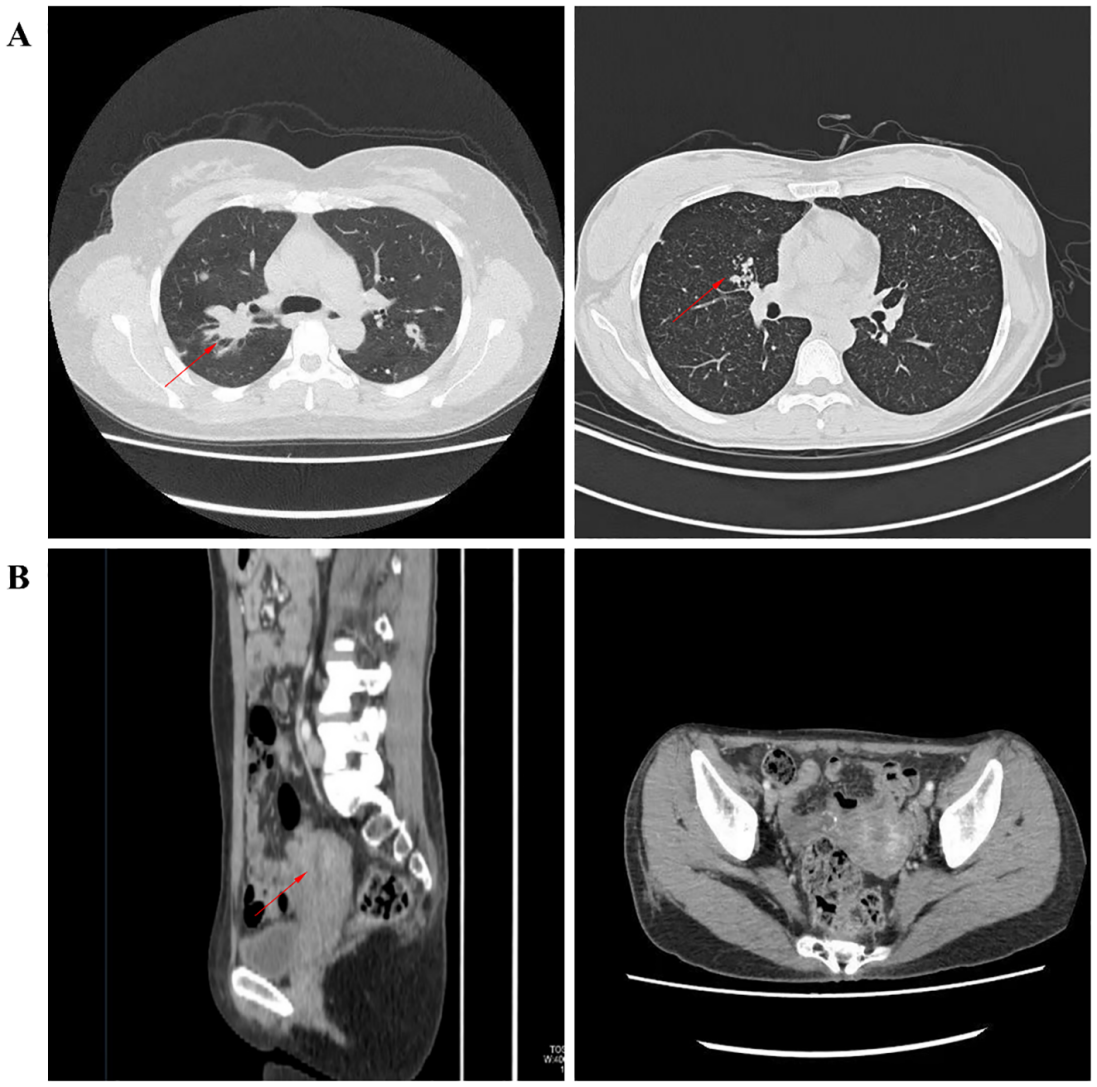
Illustrates imaging findings. **(A)** depicts nodular and patchy high-density shadows in the lungs, unevenly distributed and indicative of tuberculous lesions. **(B)** presents pelvic CT images in axial and sagittal planes, showing the uterus and surrounding structures.

The control group consisted of 10 female pulmonary tuberculosis (PTB) patients with a mean age of 44.4 years. The participants’ ages ranged from 20 to 75 years. The endometrial tuberculosis (ETB) group included 10 female patients with a mean age of 40.4 years, among whom 7 had a history of PTB, 1 had a history of spinal tuberculosis, and 2 had no documented history of tuberculosis. The participants’ ages ranged from 20 to 65 years. There were no significant differences between the two groups in peripheral blood parameters, including white blood cells, red blood cells, hemoglobin, monocytes, lymphocytes, neutrophils, eosinophils, and basophils.In the PTB group, chest CT scans revealed structural and pathological changes in the lungs. The images showed nodular and patchy high-density shadows distributed unevenly, indicative of tuberculous lesions. In contrast, pelvic CT scans of the ETB group depicted the uterus and surrounding structures without notable abnormalities. Compared to PTB imaging, CT findings for ETB were less pronounced. (**Table 1**; **Figure 2**).

**Table 1:** Presents a comparative analysis of clinical data between the pulmonary tuberculosis (PTB) group and the endometrial tuberculosis (ETB) group.

	Pulmonary tuberculosis(n=10)	Endometrial tuberculosis(n=10)	P value
Gender (Male)	n=10	n=10	/
TB Type (MTB)	n=10	n=10	/
Age (Year)	$\bar{x}$ =44.4	$\bar{x}$ =40.4	0.594
White blood cell	$\bar{x}$ =5.928	$\bar{x}$ =5.159	0.339
Red blood cell	$\bar{x}$ =4.414	$\bar{x}$ =4.137	0.211
Hemoglobin	$\bar{x}$ =124.200	$\bar{x}$ =116.100	0.154
Monocyte	$\bar{x}$ =0.383	$\bar{x}$ =0.319	0.315
Lymphocyte	$\bar{x}$ =1.389	$\bar{x}$ =1.284	0.638
Neutrophils	$\bar{x}$ =3.329	$\bar{x}$ =3.677	0.518
Eosinophilic granulocyte	$\bar{x}$ =0.115	$\bar{x}$ =0.103	0.685
Basophil granulocyte	$\bar{x}$ =0.031	$\bar{x}$ =0.020	0.153

### 3.2 Pathological features

**Figure 3:**
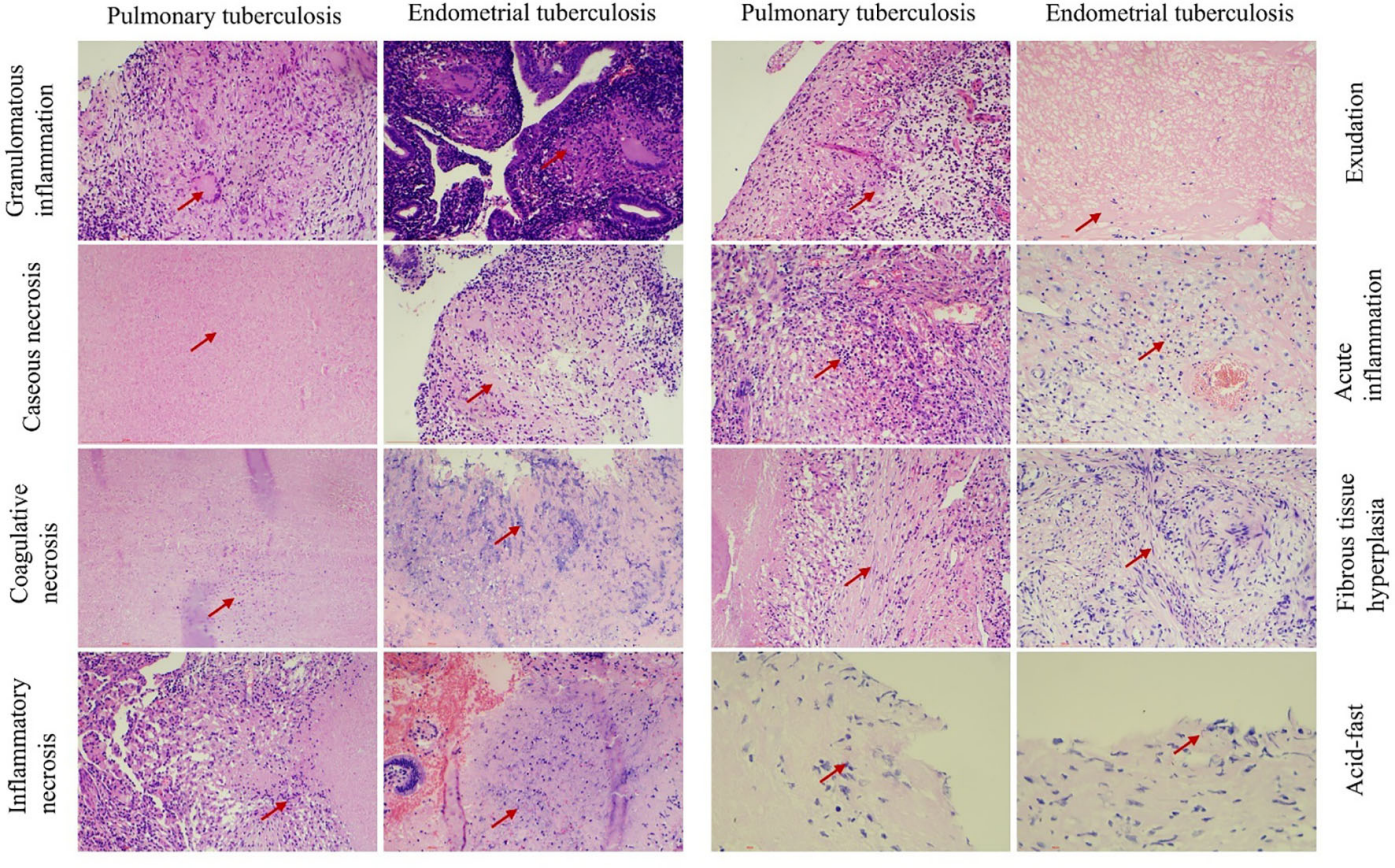
Illustrates the pathological features observed in both groups. Chronic granulomatous inflammation with caseous necrosis is a hallmark pathological change in pulmonary tuberculosis (PTB) and endometrial tuberculosis (ETB). Hematoxylin and eosin (H&E) staining reveals coagulation necrosis,inflammatory necrosis, exudation, acute inflammation, and fibrous tissue proliferation. Acid-fast staining identifies the presence of *Mycobacterium tuberculosis*. Scale bar: 200 μm; magnification: H&E (200×).

In terms of detection methods, molecular testing yielded positive results in all cases. Acid-fast staining analysis revealed a positive rate of 30% in the PTB group, compared to 10% in the ETB group. This result indicates that the PTB group exhibited a significantly higher positive response to acid-fast staining than the ETB group, suggesting a distinct pathological difference between the two groups. Among the PTB cases, 5 patients (50%) exhibited typical granulomatous lesions, 70% of which were accompanied by caseous necrosis. Additionally, coagulative necrosis (50%), acute inflammation (40%), exudation (50%), inflammatory necrosis (30%), and fibrous tissue hyperplasia (60%) were observed in some PTB cases. No abscesses were detected in this group.In the ETB group, 8 patients (80%) displayed typical granulomatous lesions, 50% of which included caseous necrosis. Furthermore, coagulative necrosis (30%), acute inflammation (40%), exudation (50%), inflammatory necrosis (40%), and fibrous tissue hyperplasia (30%) were noted in some cases. The occurrence of abscesses was 10% in the ETB group. Statistical analysis revealed no significant differences between the two groups regarding the proportions of granulomatous lesions, caseous necrosis, coagulative necrosis, inflammatory necrosis, exudation, acute inflammation, fibrous tissue hyperplasia, and abscess formation. These findings suggest that different tissue types exhibit similar pathological responses to tuberculosis infection (**Table 2**; **Figure 3**).

**Table 2:** Compares the frequency of various pathological features between the pulmonary tuberculosis (PTB) group and the endometrial tuberculosis (ETB) group.

	Pulmonary tuberculosis (n=10)	Endometrial tuberculosis (n=10)	P value
Methods			
Acid-fast (+)	30% (n=3)	10% (n=1)	0.582
PCR (+)	100% (n=10)	100% (n=10)	/
Pathology			
Granulomatous inflammation	50% (n=5)	80% (n=8)	0.350
Caseous necrosis	70% (n=7)	50% (n=5)	0.650
Coagulative necrosis	50% (n=5)	30% (n=3)	0.650
Inflammatory necrosis	30% (n=3)	40% (n=4)	1.000
Exudation	50% (n=5)	50% (n=5)	1.000
Acute inflammation	40% (n=4)	40% (n=4)	1.000
Fibrous tissue hyperplasia	60% (n=6)	30% (n=3)	0.370
Abscess	0% (n=0)	10% (n=1)	1.000

### 3.3 Immune cell infiltration

**Figure 4:**
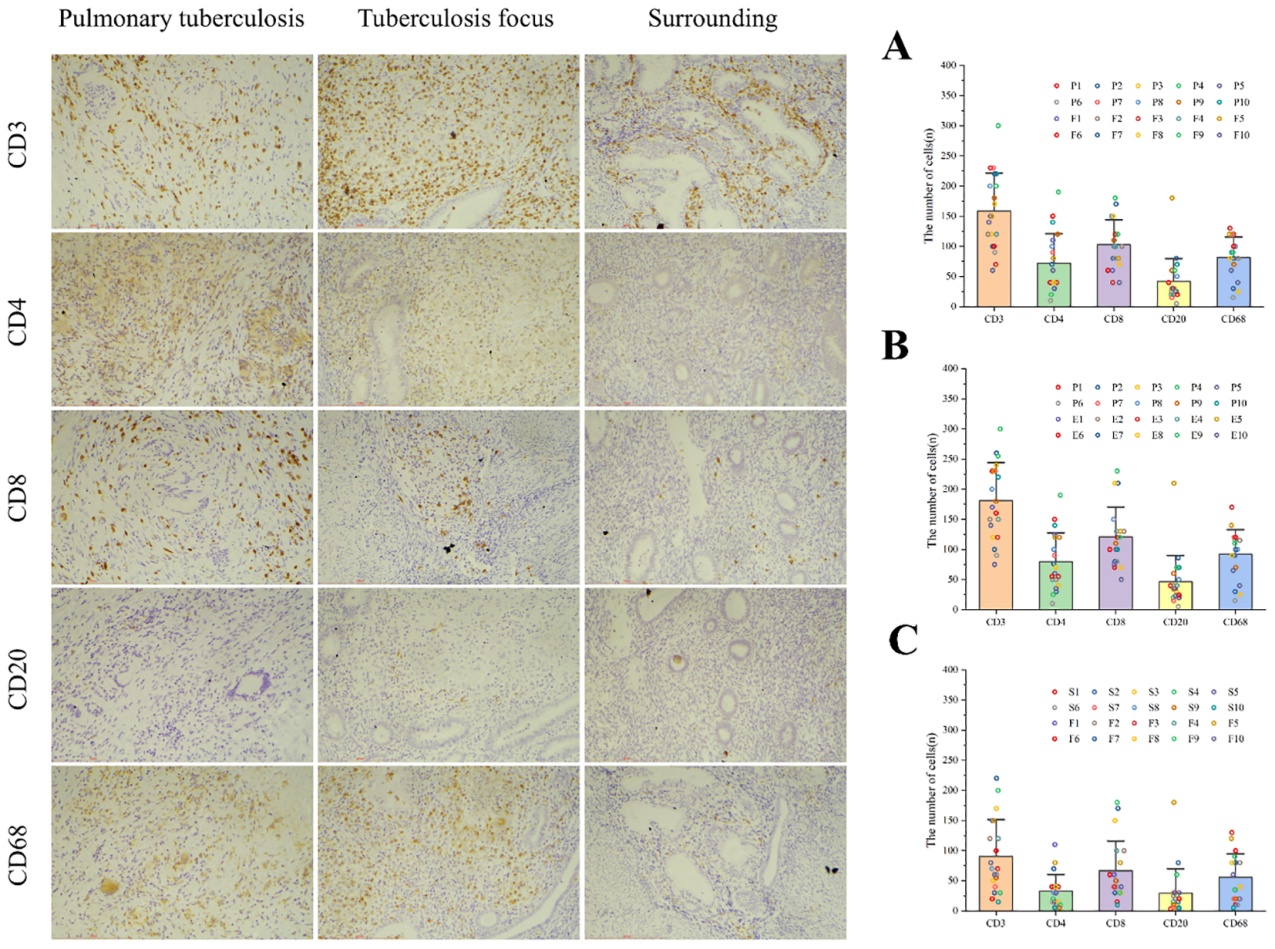
Illustrates immune cell infiltration and statistical analysis among the three groups. Immune cells, including CD3+ T cells, CD4+ T cells, CD8+ T cells, CD20+ B cells, and CD68+ macrophages, were identified using immunohistochemical staining. **(A)** Comparison between the PTB group (P) and the ETB lesion group (F). **(B)** Comparison between the PTB group (P) and the overall ETB group (E). **(C)** Comparison between the ETB lesion group (F) and its surrounding tissues (S). Scale bar: 200 μm; magnification: immunohistochemical staining (200×).

Since CD3+ represents the total T cell population, this marker was used to assess overall T cell infiltration in the endometrium, while the CD4+ and CD8+ subpopulations were employed to evaluate specific immune response patterns. Immunohistochemical analysis revealed no statistically significant differences in the counts of CD3+ T cells, CD4+ T cells, CD8+ T cells, CD20+ B cells, and CD68+ macrophages between the pulmonary tuberculosis (PTB) and endometrial tuberculosis (ETB) lesion areas. However, further comparison within the ETB group demonstrated that the numbers of CD3+ T cells, CD4+ T cells, CD8+ T cells, CD20+ B cells, and CD68+ macrophages were significantly higher in lesion areas compared to surrounding tissues. (**Table 3**; **Figure 4**).

**Table 3:** Presents a comparative analysis of immune cell differences among the pulmonary tuberculosis (PTB) group, the endometrial tuberculosis (ETB) group, and the surrounding tissues of ETB lesions.

	Pulmonary tuberculosis VS Tuberculosis focus			Pulmonary tuberculosis VS Endometrial tuberculosis			Surrounding VS Tuberculosis focus		
	Pulmonary tuberculosis( $\bar{x}$ )	Tuberculosis focus ( $\bar{x}$ )	P	Pulmonary tuberculosis( $\bar{x}$ )	Endometrial tuberculosis ( $\bar{x}$ )	P	Surrounding ( $\bar{x}$ )	Tuberculosis focus ( $\bar{x}$ )	P
CD3	181	136	0.113	181	181	1.000	45	136	<0.001
CD4	93	51	0.053	93	66.1	0.220	15.1	51	0.001
CD8	108	98	0.600	108	133.5	0.262	35.5	98	0.002
CD20	33.5	50	0.345	33.5	59.6	0.188	9.6	50	0.021
CD68	73	90	0.272	73	111.5	0.029	21.5	90	<0.001

### 3.4 Expression of inflammatory factors

**Figure 5:**
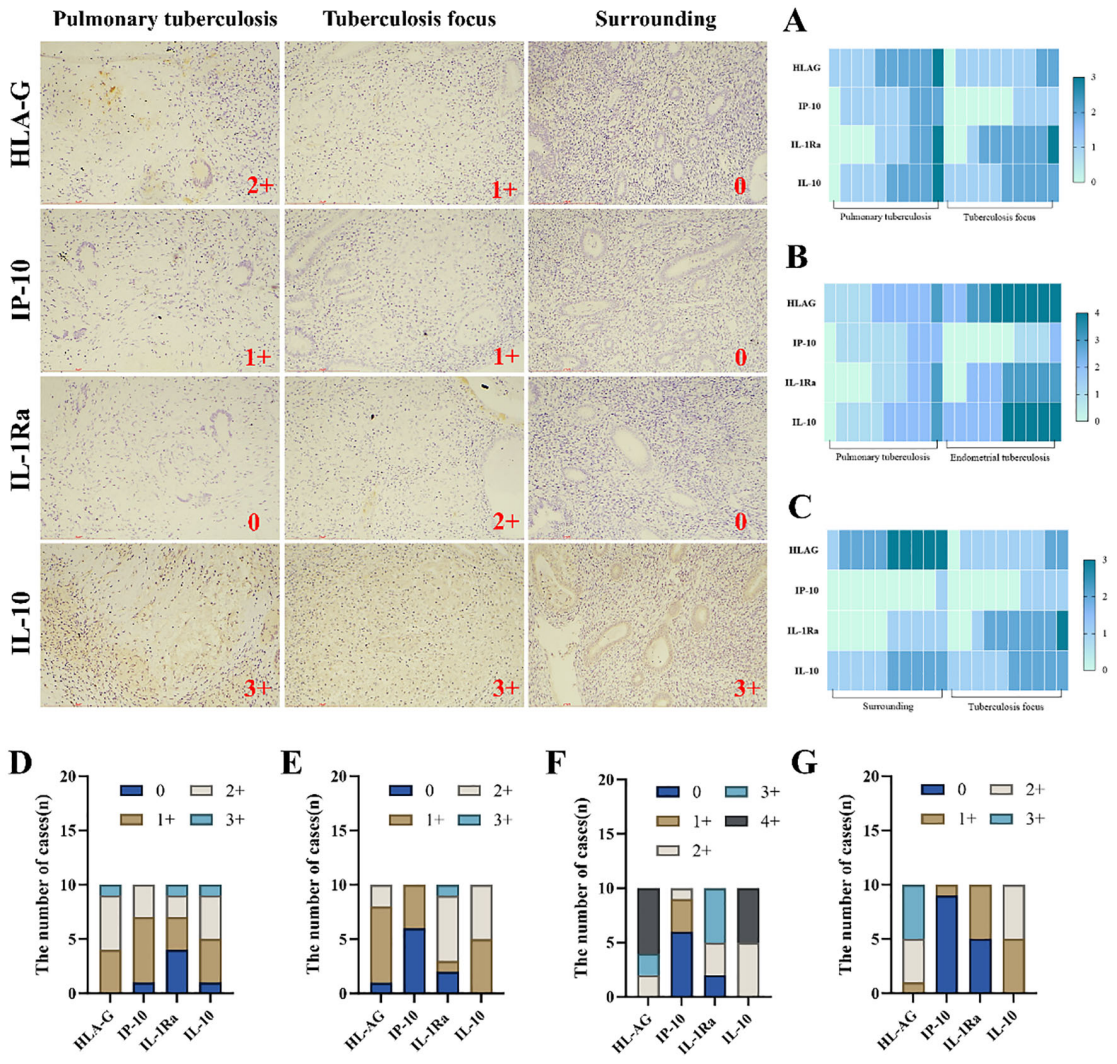
Illustrates the infiltration and expression of inflammatory factors across three groups. Immunohistochemical staining was employed to visualize the infiltration of inflammatory factors. **(A)** Comparison between the PTB group and the ETB lesion group. **(B)** Comparison between the PTB group and the overall ETB group. **(C)** Comparison between the ETB lesion group and its surrounding tissues. **(D)** The PTB group. **(E)** The ETB lesion group. **(F)** The overall ETB group. **(G)** surrounding tissues. Scale bar: 200 μm; magnification: 200×, immunohistochemical staining.

In this study, immunohistochemical staining intensity was assessed in a blinded manner by three associate chief pathologists. In cases of discrepancy, the final score was determined through joint discussion among the three pathologists to minimize observer bias and ensure result consistency. HLA-G, IP-10, IL-1Ra, and IL-10 play critical roles in maintaining normal pregnancy, and their dysregulated expression is a significant factor contributing to infertility. Immunohistochemical analysis further revealed that the expression levels of HLA-G and IP-10 were significantly higher in the lesion areas of the endometrial tuberculosis (ETB) group compared to pulmonary tuberculosis (PTB) patients. Additionally, HLA-G, IP-10, IL-1Ra, and IL-10 showed markedly elevated expression in the ETB group compared to the PTB group. Within the ETB group, HLA-G and IL-1Ra exhibited significantly higher expression in lesion areas than in surrounding tissues. (**Table 4**; **Figure 5**).

**Table 4:** presents a comparative analysis of inflammatory factor expression among the pulmonary tuberculosis (PTB) group, the endometrial tuberculosis (ETB) group, and the surrounding tissues of ETB lesions.

	Pulmonary tuberculosis VS Tuberculosis focus								
	Pulmonary tuberculosis				Tuberculosis focus				P
	0	1+	2+	3+	0	1+	2+	3+	
HLA-G	0	4	5	1	1	7	2	0	0.051
IP-10	1	6	3	0	6	4	0	0	0.010
IL-1Ra	4	3	2	1	2	1	6	1	0.176
IL-10	1	4	4	1	0	5	5	0	1.000

	Pulmonary tuberculosis VS Endometrial tuberculosis										
	Pulmonary tuberculosis					Endometrial tuberculosis					P
	0	1+	2+	3+	4+	0	1+	2+	3+	4+	
HLA-G	0	4	5	1	0	0	0	2	2	6	<0.001
IP-10	1	6	3	0	0	6	3	1	0	0	0.031
IL-1Ra	4	3	2	1	0	2	0	3	5	0	0.046
IL-10	1	4	4	1	0	0	0	5	0	5	0.005

	Surrounding VS Tuberculosis focus								
	Surrounding				Tuberculosis focus				P
	0	1+	2+	3+	0	1+	2+	3+	
HLA-G	0	1	4	5	1	7	2	0	0.001
IP-10	9	1	0	0	6	4	0	0	0.131
IL-1Ra	5	5	0	0	2	1	6	1	0.010
IL-10	0	5	5	0	0	5	5	0	1.000

## 4 Discussion

Pulmonary tuberculosis (PTB) and endometrial tuberculosis (ETB) are distinct forms of tuberculosis infection caused by *Mycobacterium tuberculosis* (Basaraba and Hunter, 2017). Despite sharing a common pathogen, the pathological manifestations of these diseases differ due to the anatomical structures and immune microenvironments of the affected sites. PTB is the most prevalent form of tuberculosis, primarily transmitted via inhalation of infectious aerosols. The dense structure of pulmonary tissue and the high concentration of immune cells in alveoli contribute to the characteristic pathological features of PTB, including granulomas, caseous necrosis, and fibrosis. These changes reflect the intense immune response triggered by the pathogen in lung tissue (Dorhoi and Kaufmann, 2016; Stek et al., 2018). In contrast, ETB is a reproductive system infection typically spread hematogenously to the endometrium. Unlike the dense pulmonary tissue, the endometrium is relatively loose in structure, enriched with glands and blood vessels. Its cyclical shedding and regeneration add complexity to the local immune environment, differentiating its pathological and immunological responses from those observed in PTB (Salamonsen et al., 2021). This study revealed that the pathological features of endometrial tuberculosis (ETB) patients include granulomatous lesions, caseous necrosis, coagulative necrosis, inflammatory necrosis, exudation, acute inflammation, fibrous tissue proliferation, and rare abscess formation. The high prevalence of granulomatous inflammation in ETB may indicate that the endometrium’s immune response is more inclined to control lesions through localized inflammation and granuloma formation. Our study revealed differences in pathological features between pulmonary tuberculosis (PTB) and ETB, although these differences did not reach statistical significance. Granulomatous inflammation was observed in 80% of ETB patients compared to 50% of PTB patients, suggesting that the endometrium may be more prone to granuloma formation. Caseous necrosis was common in both groups, occurring in 70% of PTB patients and 50% of ETB patients. Although the differences were not statistically significant, these variations may stem from structural and immunological differences between lung tissue and the endometrium.In our study, the incidence of abscess formation in ETB patients was low (10%), while no abscesses were detected in PTB patients. The immune microenvironment of the endometrium may favor granuloma formation as a mechanism to combat infection. Due to its rich vascularization and glandular composition, the endometrium may exhibit a more robust local inflammatory response to tuberculosis infection, occasionally accompanied by limited abscess formation. These findings suggest that, while tuberculosis lesions trigger similar pathological responses across different tissues, the unique anatomical structure and immune properties of the endometrium contribute to its distinct pathological features.

Immune cells play a critical role in infertility by maintaining immune balance within the endometrium under normal conditions, facilitating embryo implantation and pregnancy (Robertson et al., 2022). However, dysfunction or overactivation of T cells (CD3, CD4, CD8), B cells (CD20), and macrophages (CD68) can disrupt endometrial immune homeostasis, triggering chronic inflammation and impairing embryo implantation, ultimately leading to infertility (Yang et al., 2019). Mycobacterium tuberculosis infection significantly activates these immune cells (Macdonald et al., 2012). The heightened activity of T cells and macrophages induced by tuberculosis infection can damage the endometrium, causing fibrosis and intrauterine adhesions that hinder embryo implantation. Furthermore, the immune imbalance caused by infection undermines the endometrium’s supportive function, rendering it unable to provide a stable environment for embryo development. Even after infection resolution, persistent endometrial damage may pose a long-term risk of infertility. In this study, no significant differences were observed in the expression of most immune cells between pulmonary tuberculosis (PTB) and endometrial tuberculosis (ETB) lesions and their surrounding tissues, except for CD68, which showed a statistically significant difference. This finding suggests that macrophages may respond differently in these two forms of tuberculosis. Macrophages play a pivotal role in tissue remodeling, fibrosis, and granuloma formation during tuberculosis infection, and their heightened expression in endometrial tuberculosis (ETB) may be associated with a more robust phagocytic and immunoregulatory function within the endometrial environment (Abengozar-Muela et al., 2020). These cells are crucial in controlling Mycobacterium tuberculosis infection; however, their activation may lead to fibrosis and intrauterine adhesions, which can impede embryo implantation. The involvement of macrophages in granuloma formation further underscores their role in modulating local inflammatory responses (Hickman et al., 2002). These findings suggest that the role of macrophages in tissue remodeling and fibrosis may contribute to the long-term reproductive consequences of ETB, including infertility. Comparisons between ETB lesions and surrounding tissues revealed significant differences in the expression of all immune cells, indicating more active immune infiltration at the site of infection. These findings further support the notion that ETB triggers a localized and robust immune response, with the infiltration and activation of immune cells potentially serving as a critical mechanism for controlling local tuberculosis infection.

Inflammatory factors play a pivotal role in preparing the endometrium for embryo implantation under normal physiological conditions. However, their overactivation can trigger chronic inflammation in the endometrium, leading to tissue damage, impaired embryo implantation, and an increased risk of infertility (Granot et al., 2012). Tuberculosis infection significantly elevates the expression of certain inflammatory factors, such as IP-10, IL-1Ra, and IL-10, thereby activating the immune system (Fisher et al., 2022). The heightened expression of these factors induces persistent local inflammation or immunosuppression, disrupting the endometrial environment and impairing reproductive function. IP-10 recruits T cells to amplify inflammation, while IL-1Ra and IL-10 attempt to suppress excessive immune responses but may inadvertently delay tissue repair (Dufour et al., 2002; Carlini et al., 2023). In chronic tuberculosis infections, HLA-G expression is upregulated to modulate inflammatory responses and prevent excessive tissue damage. Studies suggest that the increased expression of HLA-G may be associated with prolonged immune activation triggered by tuberculosis infection (Wang et al., 2024).Endometrial tuberculosis alters the balance of inflammatory factors, rendering the endometrium less capable of supporting normal embryo development. Prolonged inflammation and immune suppression may lead to implantation failure or early pregnancy loss, increasing the risk of adverse pregnancy outcomes. This study focuses on the expression of four key inflammatory markers: HLA-G, IP-10, IL-1Ra, and IL-10. The expression of HLA-G showed significant differences between pulmonary tuberculosis (PTB) and endometrial tuberculosis (ETB) lesion and peri-lesion tissues. Furthermore, notable differences were also observed between the ETB lesion areas and their surrounding tissues. HLA-G is an immunoregulatory molecule that plays a critical role in maternal-fetal tolerance. It modulates immune responses to prevent excessive inflammation and tissue damage (Yang et al., 2022). Our study found that HLA-G expression is elevated in ETB lesions, which may contribute to the establishment of local immune tolerance and the suppression of excessive immune responses to *Mycobacterium tuberculosis* infection. This mechanism may help prevent tissue damage while simultaneously controlling infection, particularly within the reproductive system. Similarly, IP-10 exhibited significant differences in expression among PTB, ETB lesions, and peri-lesion tissues, suggesting distinct functional roles in these infections. IP-10, a chemokine, recruits T cells and natural killer cells to the infection site, and its differential expression likely reflects variations in immune cell recruitment in tuberculosis infections affecting different organs (Liu et al., 2000).IL-1Ra and IL-10 were primarily associated with immunosuppressive functions. The elevated expression of IL-1Ra in ETB lesion and peri-lesion tissues, as well as the observed differences between these regions, underscores the importance of this suppressive factor in ETB pathogenesis. IL-1Ra is an anti-inflammatory cytokine essential for regulating immune responses and limiting inflammation (Domingo-Gonzalez et al., 2016). The increased expression of IL-1Ra in ETB lesions suggests its pivotal role in modulating local immune responses, reducing excessive inflammation, and preventing tissue damage. IL-1Ra exerts its effects by inhibiting IL-1 activity, thereby regulating the inflammatory response. Its upregulation in ETB may serve as a mechanism to control local inflammation and mitigate excessive tissue destruction. The balance between pro-inflammatory and anti-inflammatory cytokines is crucial for maintaining reproductive health, and disruption of this balance may contribute to infertility, highlighting the critical role of immune regulation in the pathogenesis of ETB. IL-10 also showed significant differential expression between PTB and ETB lesions, highlighting stronger immunosuppressive signaling in ETB patients.

In conclusion, endometrial tuberculosis (ETB) exhibit distinct immunological features compared to pulmonary tuberculosis (PTB), particularly in the expression of macrophages CD68, HLA-G, and IL-1Ra. The elevated expression of CD68 suggests that macrophages play a crucial role in the immune response to ETB. The upregulation of HLA-G and IL-1Ra further highlights the suppressive pathways utilized by ETB to modulate local immune responses, maintaining immune balance and preventing excessive immune-mediated tissue damage. The significant differences observed between ETB lesions and surrounding tissues underscore the intensity of local immune reactions. In ETB lesions, the infiltration and activation of immune cells are critical mechanisms for combating tuberculosis infection. However, such robust localized immune responses may result in chronic inflammation, exacerbating tissue damage and potentially contributing to the risk of infertility associated with ETB.

Although this study provides an in-depth analysis of the pathological features of endometrial tuberculosis, certain limitations remain. First, the small sample size may restrict the generalizability and representativeness of the findings. Secondly, our study population ranged in age from 20 to 65 years, and advancing age is known to impact reproductive capacity, primarily through diminished regenerative potential and reduced immune tolerance (Gruhn et al., 2019). In patients with endometrial tuberculosis (ETB), advanced maternal age may exacerbate endometrial fibrosis and chronic inflammation, further compromising embryo implantation and pregnancy success rates. Additionally, aging is associated with diminished ovarian reserve, reduced endometrial receptivity, and an increased risk of pregnancy complications, all of which may further exacerbate infertility in ETB patients (White et al., 2022). Due to the limited sample size, this study did not perform an age-stratified analysis. We acknowledge this limitation and recommend future studies with larger cohorts to comprehensively evaluate the impact of maternal age on reproductive outcomes in ETB patients. Furthermore, the hormonal cycle of the endometrium may influence immune responses and ETB progression. Hormonal fluctuations throughout the menstrual cycle may alter immune cell infiltration and tissue remodeling, thereby affecting the course of tuberculous infection (Cunningham and Gilkeson, 2011). Future research should consider these hormonal variations and incorporate larger sample sizes to validate these findings. Finally, although a variety of immune cells and antibody markers were employed, other potential molecules and immune mechanisms have not been explored. We ensured data robustness by employing rigorous statistical methods, which support the reliability of our findings. Future research should focus on increasing the sample size to further validate the roles and mechanisms of these immune cells and molecules. Additionally, given the close association between endometrial tuberculosis and infertility, future studies could investigate the role of immune regulatory factors in infertility, potentially offering new therapeutic targets for clinical treatment.

## 5 Conclusions

The pathological characteristics of endometrial tuberculosis (ETB) and pulmonary tuberculosis (PTB) do not show significant differences; however, notable distinctions are observed in the expression of immune cells and inflammatory factors, particularly macrophages, HLA-G, IP-10, and IL-1Ra. The infiltration of immune cells and the elevated expression of immunoregulatory factors suggest that ETB employs localized immune responses to control infection. However, this process may also contribute to chronic inflammation and tissue damage. This study provides critical foundational data for understanding the immunological mechanisms of ETB and its association with infertility.

## Data Availability

The original contributions presented in the study are included in the article/supplementary material. Further inquiries can be directed to the corresponding author/s.
